# Quantum mechanical modeling of interstellar molecules on cosmic dusts: H_2_O, NH_3_, and CO_2_

**DOI:** 10.3389/fchem.2022.1040703

**Published:** 2022-11-10

**Authors:** Fangfang Li, Donghui Quan, Xia Zhang, Xiaohu Li, Jarken Esimbek

**Affiliations:** ^1^ Xinjiang Astronomical Observatory, Chinese Academy of Sciences, Urumqi, China; ^2^ School of Astronomy and Space Science, University of the Chinese Academy of Sciences, Beijing, China; ^3^ Zhejiang Laboratory, Research Center for Intelligent Computing Platforms, Hangzhou, China; ^4^ Department of Chemistry, Eastern Kentucky University, Richmond, KY, United States; ^5^ Xinjiang Key Laboratory of Radio Astronomy, Urumqi, China; ^6^ Key Laboratory of Radio Astronomy, Chinese Academy of Sciences, Urumqi, China

**Keywords:** astrochemistry, theory, interstellar: matter, interstellar: abundances, modeling

## Abstract

Since the first detection of CH molecule in interstellar medium (ISM), more than 270 molecules have been identified in various astronomical sources in ISM. These molecules include big complex ones, such as fullerene (C_60_) and polycyclic aromatic hydrocarbons (PAHs), which are the main components of carbonaceous dust. Dust surface chemistry plays an important role in explaining the formation of interstellar molecules. However, many of the dust surface chemical parameters, such as the adsorption energies, are still of uncertainty. Here we present a study of the adsorption of water (H_2_O), ammonia (NH_3_), and carbon dioxide (CO_2_) on graphene-like substrate within the framework of density functional theory (DFT). We used Gaussian 16 software and adopted the corrected generalized gradient approximation (GGA) with the Perdew–Burke–Ernzerhof (PBE) functions. We determined the optimal accretion position of the studied molecules on the graphene-like surface and calculated the adsorption energies. Furthermore, according to the density of states and molecular orbitals of the adsorbed states, we analyzed the charge transfer between the molecules and the graphene-like surface. These results can provide more accurate parameters for calculating the chemical reaction rates on the dust surface, thus contributing to the understanding of dust-surface reactions in ISM.

## 1 Introduction

The finding of interstellar molecules is one of the four major discoveries of astronomy in the 1960’s. Astronomers used large radio telescopes and detected NH_3_ and H_2_O in the central region of the Milky (Cheung et al., 1968; Cheung et al., 1969). These molecules are abundant behind the dust clouds, forming massive “molecular clouds”. Soon, astronomers discovered a more complex organic molecule, formaldehyde (H_2_CO) (Snyder et al., 1969). It is widespread, not only in the central region of the Milky, but also in the Orion Nebula and other regions. Since then, more interstellar molecules have been found in the space, including inorganic and organic ones. For example, hydroxyl (−OH), carbon monoxide (CO), carbon dioxide (CO_2_), hydrogen cyanide (HCN), methyl alcohol (CH_3_OH), acetaldehyde (CH_3_CHO), cyanoacetylene (HC_3_N), methylamine (CH_3_NH_2_), etc. To date, more than 270 molecules and ions have been detected in interstellar space^1^.

Carbon-containing organic complex molecules are the basic materials for the origin of life. Carbonaceous dust is one of the main components of interstellar dust, and its infrared spectrum is an important indicator for detecting the physical and chemical conditions of various celestial environments (Draine et al., 2003). Carbonaceous dust such as graphite, nanodiamond, PAHs, C_60_, hydrogenated amorphous carbon and other dust particles are important research objects of interstellar medium (Li et al., 2020). Indeed, many of these carbon allotropes have been found in the ISM Some of them are indicated from pre-solar grains isolated from carbonaceous primitive (such as nanodiamond, graphite; see Lewis et al., 1987; Amari et al., 1990), and some are from the observations of molecular and solid-state features in astronomical spectra (such as amorphous carbon, PAHs; see Stecher and Donn 1965; Leger and Puget 1984). After that, the detection of C_60_ and C_70_ and their ions in the interstellar and circumstellar space has been reported based on their characteristic vibrational spectral bands in infrared (Cami et al., 2010; Strelnikov et al., 2015).

The formation process and chemical evolution of interstellar molecules are not completely understood due to their diversity and complexity. Astronomers wonder how and when interstellar molecules form in clouds as gases and solids. How do interstellar molecules generate, survive, and further evolve on the surface of interstellar dust, under the extremely low temperature and ultra-high vacuum conditions associated with interstellar clouds? They could be formed in the gas phase, on the surface of bare dust particles, or in the ice mantles that cover bare grains in cold dense interstellar clouds (Herbst 2013; Herbst 2014). At low temperatures, only the exothermic and barrier-free reactions (or reactions with low potential barriers) can occur in the gas phase in dense clouds. Thus, free radical reactions and ion-molecule reactions dominate gas phase chemistry (Herbst 1973; Watson 1973). Atoms and molecular ions are produced by cosmic rays (including photons). At the same time, it is recognized that large numbers of species and organic molecules in the gas phase cannot be produced efficiently. When the dense cloud is sufficiently cooled (≤20 K), the gaseous atoms (e.g., H, C, N, O) and molecules (e.g., H_2_ and CO) are deposited on the dust surface, and the new molecules are formed through the cold surface chemical reaction, due to the thermal fluctuations on the dust surface, the matter can be desorbed to the gas phase again (Hama and Watanabe, 2013; Wakelam et al., 2017a).

Some important molecules, such as H_2_O, CO_2_, NH_3_, and organic molecules, require surface reactions to attain the observed abundances. In addition, H_2_O, CO_2_, NH_3_ molecules are also the main components of the ice mantles covered on the dust grains (Hama and Watanabe, 2013). Currently, the rate coefficients of gas phase reactions in chemical reaction networks are mostly estimated. Experimental and computational quantum chemistry are needed to measure/calculate these reaction rates more accurately in the future (Herbst, 2013). The dust surface chemistry model is even more uncertain, and current ones do not take into account all the important physical processes on dust. The essential parameters, such as desorption energies, also have great uncertainty (Chen et al., 2020). All these require more accurate measurements of experimental and/or theoretical astrochemical studies.

In recent years, astronomers have calculated and studied the binding energy of species on interstellar various substrates (Wakelam et al., 2017b; Duflot et al., 2021; Bovolenta et al., 2022; Perrero et al., 2022; Tinacci et al., 2022). In this study, we set up models of interstellar molecules H_2_O, NH_3,_ and CO_2_ landing on graphene-like surfaces through quantum chemistry computation and calculated their adsorption energies. In order to determine the changes in the electrical properties of graphene-like surfaces before and after the adsorption H_2_O, NH_3_ and CO_2_, we studied their density of states (DOS) on the graphene-like surface. In Section 2, we introduce the calculation method. We discuss the results in Section 3 and summarize the major findings in Section 4.

## 2 Calculation method and details

Based on the DFT (Delley 1990; Ni et al., 2020), we used PBE method with the 6-31G (d, p) basis set to optimize the interstellar molecule H_2_O, NH_3_, CO_2_ and graphene-like structures. The graphene-like surface contains 48 carbon atoms, and H is added to the edge to eliminate the edge effect. In addition, the PBE functional was mostly used for 2D material correlation calculations (Li, et al., 2022). Considering that PBE amplification underestimates the non-local interaction, the PBE0-D3 method (Mehta et al., 2018) was adopted to calculate the adsorption energies and the DOS, including the total (TDOS), and partial (PDOS) density of states. All quantum chemical calculations were run with the GAUSSIAN 16 program package (Frisch, 2016).

**FIGURE 1:**
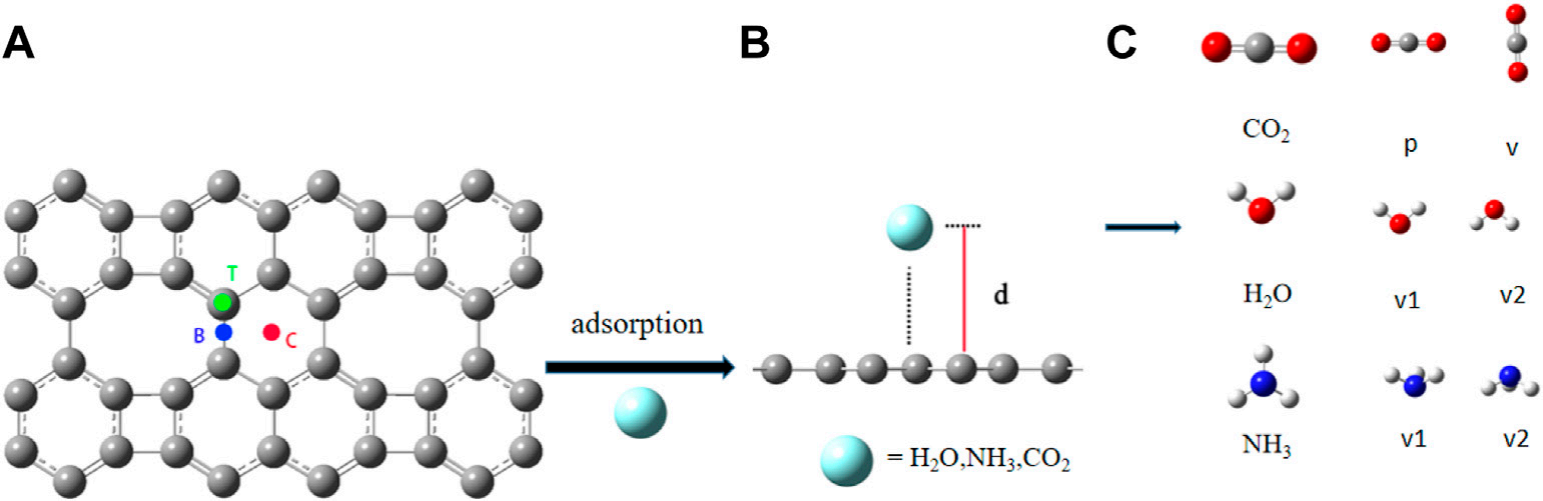
**(A)** Three adsorption sites on the graphene-like surface; **(B)** The scheme of adsorption of a molecule on the surface; **(C)** The adsorption configurations of H_2_O, NH_3_ and CO_2_.

For each of the molecules, we considered three adsorption sites on graphene-like surfaces (See Figure 1A), namely, top location (above a carbon atom, T), bridge location (the midpoint of a carbon-carbon bond, B), and center location (the center of a carbon hexagon, C). The scheme of adsorption of a molecule on the surface is shown in Figure 1B, where d is the adsorption distance between the molecule and the graphene-like surface. According to the structure of the molecule, H_2_O has two forms: the oxygen atom pointing vertically to the graphene-like surface (denoted by v1), and two hydrogen atoms pointing vertically to the graphene-like surface (denoted by v2). Coupled with the three graphene adsorption sites, so H_2_O has six adsorption modes; For NH_3_, there are two forms, the nitrogen atom pointing vertically to the surface (denoted by v1) and the three hydrogen atoms pointing vertically (denoted by v2), with a total of six adsorption modes; For CO_2_, there are two forms: parallel (denoted by p) and vertical (denoted by v), so it has six modes. The different forms of these molecules are shown in Figure 1C. Gray, red, white, and blue spheres represent carbon, oxygen, hydrogen, and nitrogen atoms, respectively.

The adsorption energy (*E*_*ads*_) is used to determine the stability of the structure before and after adsorpting interstellar molecules, and the equation is:

$$E_{ads}=E_{gr+\text{mol}}-\left(E_{gr}+E_{mol}\right)$$

where *E*_*gr*_ is the energy of graphene-like surface, *E*_*mol*_ is the energy of interstellar molecules H_2_O, NH_3_, and CO_2_, and *E*_*gr+mol*_ is the energy of the whole adsorption system. According to this definition, when the adsorption energy is negative, the adsorption process is exothermic, and the larger the absolute value of adsorption energy, the stronger the adsorption of molecules on the graphene-like surface and the more stable the configuration; On the other hand, when the adsorption energy is positive, the process is endothermic, and the larger the value, the weaker the adsorption. (Saha and Chowdhury, 2011). Moreover, when the absolute value of adsorption energy is more than 0.8 eV, it is chemical adsorption. Otherwise it is physical adsorption (Li, et al., 2022). The energy involved in the equation of *E*_*ads*_ is the electronic energy of the system, without considering the influence of temperature. If the thermodynamic properties of the system are to be further analyzed, the Gibbs free energy should be calculated, and the effects of temperature and pressure should be considered (Buelna-Garcia et al., 2020; Buelna-Garcia et al., 2021; Buelna-Garcia et al., 2022; Castillo-Quevedo et al., 2021; Wesslén et al., 2014; Pracht and Grimme, 2021; Baletto and Ferrando, 2005).

## 3 Results and discussion

**FIGURE 2:**
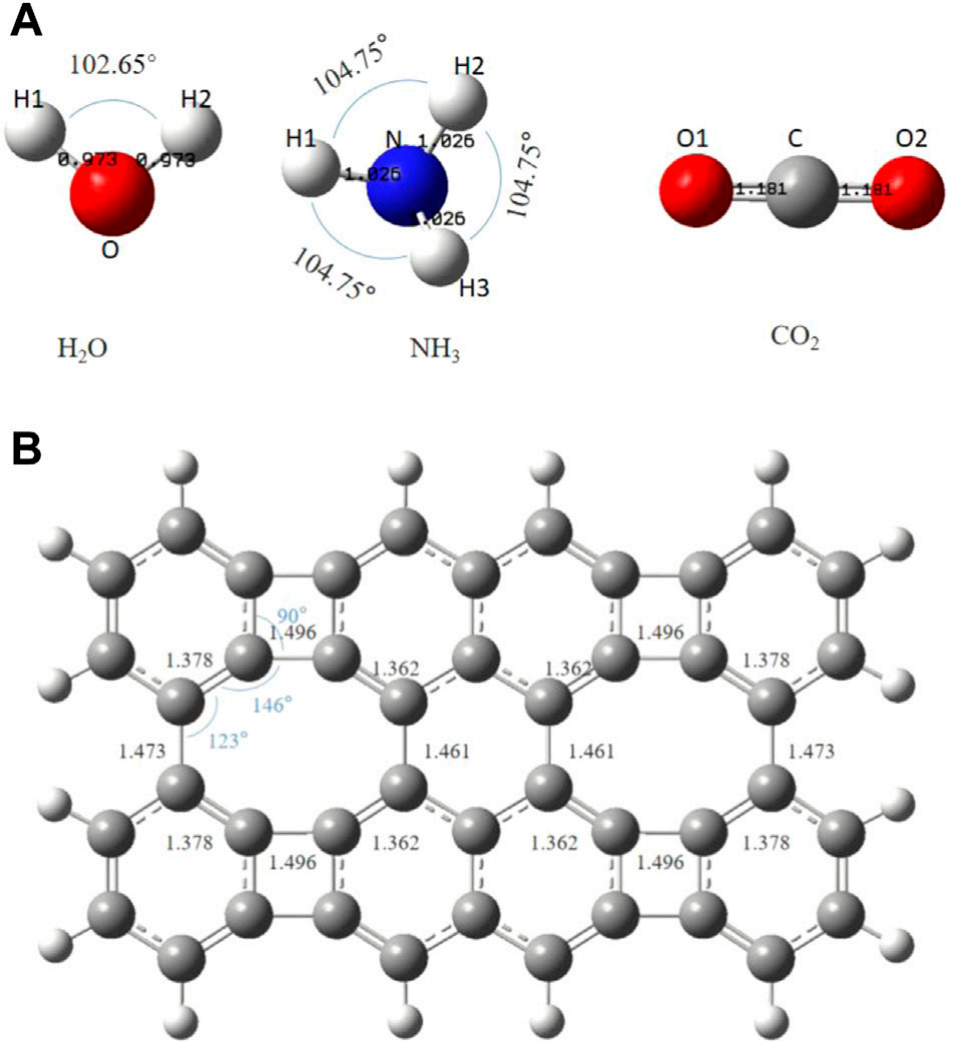
The optimized Structures of **(A)** H_2_O, NH_3_, CO_2_ and **(B)** the graphene-like surface. The bond lengths are in angstroms.

The optimized H_2_O, NH_3_, CO_2,_ and graphene-like surfaces are shown in Figure 2A. H_2_O molecule is a planar triangle, with bond lengths of 0.973Å, and the bond angle of H-O-H is 102.65°; NH_3_ molecule is a triangular cone with bond lengths of 1.026Å and a bond angle of 104.75°; CO_2_ molecule is linear with bond lengths of 1.181Å and the bond length is 180°. As it can be seen from Figure 2B, the overall C-C bond length of the graphene-like surface is 1.352–1.496Å. The C-C bonds at the non-hexagonal rings are shortened or lengthened due to the missing of hexagonal symmetry (Peng and Ahuja, 2008) The C-C-C angles range from 90° to 146°, consistent with previous studies (Liu et al., 2017).

For the adsorption of H_2_O, NH_3,_ and CO_2_ on graphene-like surfaces, we have optimized the structure of different configurations, and calculated the intrinsic vibrational frequencies. The results show that each adsorption system has no virtual frequencies, indicating that its adsorption structure is stable.

The DOS analysis can provide basic information on the effects of the electronic properties of graphene-like surfaces, which adsorbed interstellar molecules H_2_O, NH_3_, and CO_2_ (Zahedi and Seif, 2011). In addition, it can also be seen that the change of conductivity and energy gap of graphene-like surfaces before and after adsorption (Leenaerts et al., 2008; Wang et al., 2018).

### 3.1 H_2_O on the graphene-like surface

We examined the following orientations of the H_2_O molecule with respect to the graphene-like surface: the oxygen atom points vertically to the surface (v1), or two hydrogen atoms point vertically to the surface (v2). Depending on the binding site (shown in Figure 1A) on the graphene-like surface, there are six different configurations of H_2_O adsorption: T-v1, T-v2, B-v1, B-v2, C-v1, and C-v2. The calculated results are shown in Table 1, where d is the adsorption distance between the molecule and the nearest atom on the graphene-like surface.

**TABLE 1:** H_2_O on the graphene-like surface: *E*_*ads*_, d and the bond length and bond angle of H_2_O.

Configurations	*E*_*ads*_(eV)	*E*_*ads*_(K)	*d*(Å)	Bond length (Å)	Bond angle (°)
				H1-O; O-H2	
T-v1	−0.3435	−3986.18	2.50	0.957; 0.957	103.61
T-v2	−0.3436	−3987.34	2.50	0.957; 0.957	103.63
B-v1	−0.2736	−3175.02	2.80	0.956; 0.956	103.95
B-v2	−0.1610	−1868.34	3.57	0.956; 0.956	103.66
C-v1	−0.1785	−2071.42	3.35	0.955; 0.955	105.05
C-v2	−0.2690	−3121.63	2.90	0.956; 0.956	103.88

**FIGURE 3:**
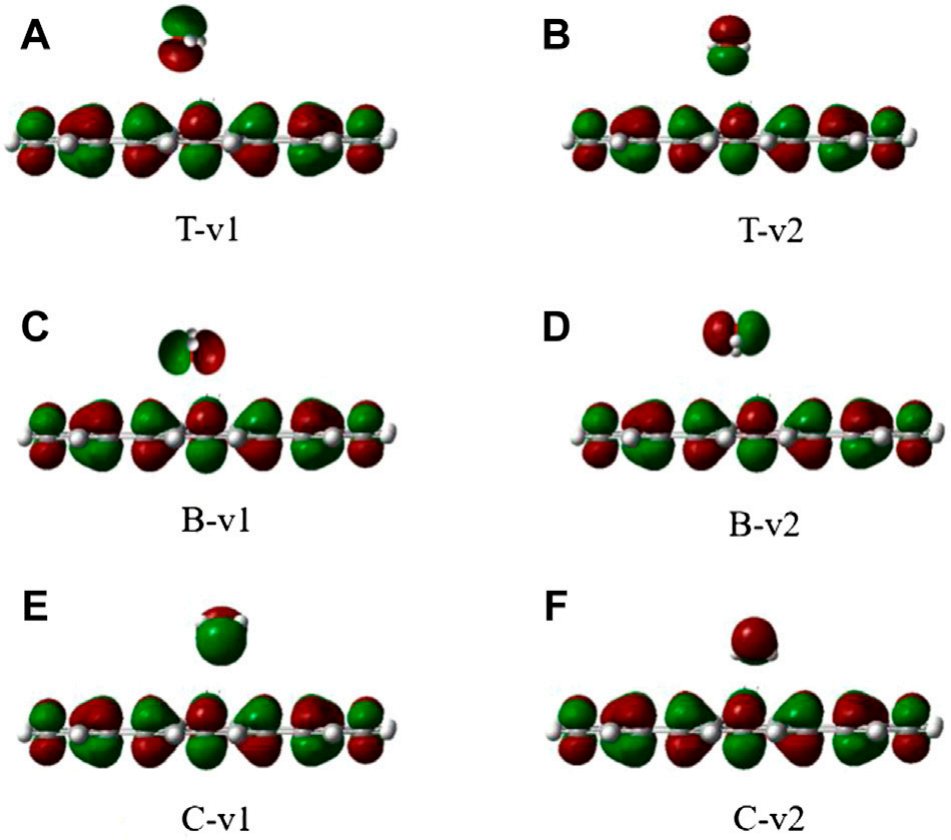
The electron density of six different configurations of H_2_O on the graphene-like surface. **(A)**: T-v1, **(B)**: T-v2, **(C)**: B-v1, **(D)**: B-v2, **(E)**: C-v1, **(F)**: and C-v2.

The stability of H_2_O on the graphene-like surface is in the order of T-v2 > T-v1 > B-v1 > C-v2 > C-v1 > B-v2, among which the highest adsorption energy is in T-v2, with a value of 0.3436eV; B-v2 has the smallest adsorption energy with the value 0.1610eV. The adsorption energy is mainly determined by the position of the binding site (C, B, T), followed by the orientation of the molecule (v, p). Furthermore, the farther the distance in between the molecule with the binding site, the smaller the adsorption energy. All the absolute value of adsorption energy in the table are less than 0.8 eV, the length of the H-O bond and the angle of H-O-H are almost unchanged. In combination with the charge density of H_2_O adsorbed on the graphene-like surface (Figure 3), it is found that there is no overlap between the electron clouds of H_2_O and the graphene-like surface, so the adsorption of H_2_O on the surface belongs to physical adsorption, indicating that there is no chemical bond between H_2_O and the surface.

**FIGURE 4:**
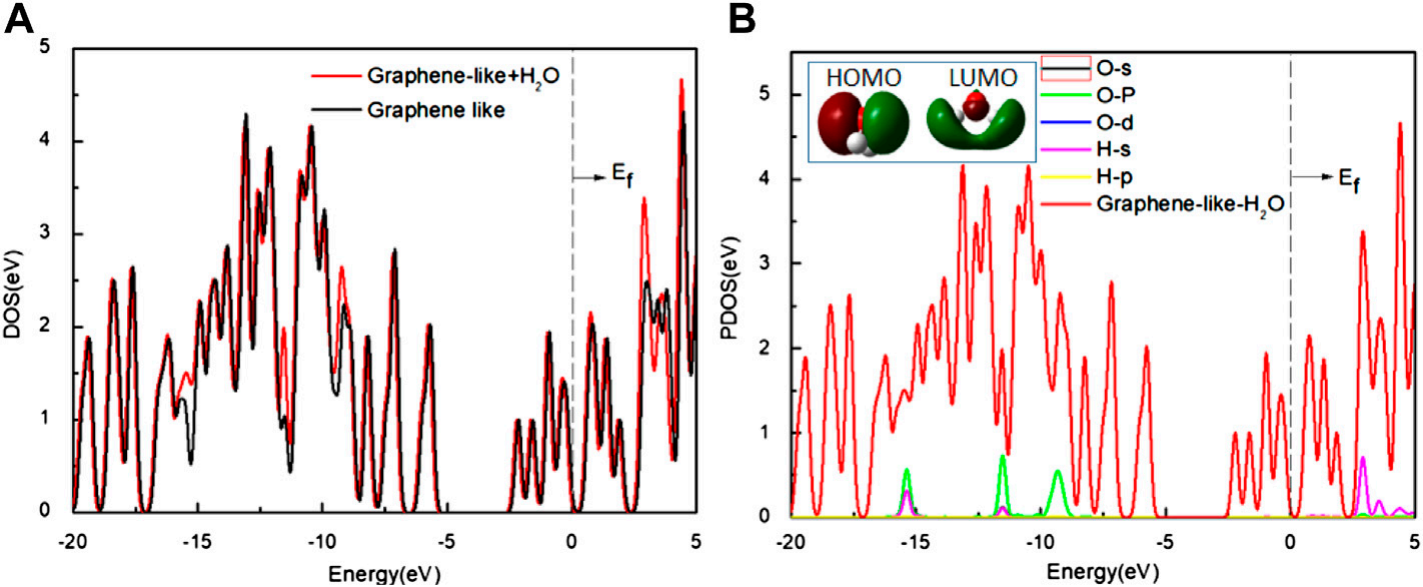
**(A)** The DOS of the graphene-like surface before and after adsorption of H_2_O; **(B)** The PDOS of H_2_O on the surface. Inset: HOMO and LUMO of H_2_O (Green and deep red lines indicate different signs of the orbital wavefunctions). Main panel: The red line is the DOS of H_2_O on the graphene-like surface, and other lines show the PDOS of the O and H atoms, respectively.

To further study the adsorption behavior, we analyzed the DOS of the graphene-like surface before and after the top-position adsorption of H_2_O (Figure 4), with the Fermi level as energy zero (Kahn 2016). There are two peaks on both sides of this zero, and the distance between the peaks is defined as the pseudogap (Loktev et al., 2001). The change of the pseudogap at a local bond reflects the change of the bond energy here. The larger the pseudogap is, the stronger chemical bonds are. On the contrary, the narrowing of the pseudogap means that the chemical bonds are easier to break.

As it can be seen from Figure 4A, the DOS of the graphene-like surface does not change significantly before and after H_2_O adsorption. No new states appear near the Fermi level. The width of the pseudogap is unchanged, indicating that H_2_O adsorption has minimal effect on graphene-like surface. Combined with the results shown in Figure 4B, it can be seen that H_2_O molecule’s HOMO is located entirely on the O atom, while LUMO is mainly located on the H atom. If the O atoms point to the graphene-like surface, HOMO plays a dominant role, but this does not induce any charge transfer because all these orbits are filled up, and Figure 4B shows that the PDOS of oxygen atoms is almost zero near the Fermi level. In the case of v2, where the H atoms point to the graphene surface, the LUMO of H_2_O interacts with the surface much more strongly, but this still does not cause any charge transfer, because all these orbits are empty. The PDOS of the H atoms near the Fermi level is almost zero.

### 3.2 NH_3_ on the graphene-like surface

For NH_3_ molecules, two orientations were studied, namely, H atoms pointing to graphene-like surface (v1), and N atoms pointing to graphene-like surface (v2). All properties are once again found to be almost invariant to the rotation around the axis perpendicular to the surface and through the nitrogen atoms. The calculated results are shown in Table 2.

**TABLE 2:** NH_3_ on the graphene-like surface: *E*_*ads*_, d and the bond length and bond angle of NH_3_.

Configurations	*E*_*ads*_(eV)	*E*_*ads*_(K)	*d*(Å)	Bond length (Å)	Bond angle (°)
				H1-N; H2-N; H3-N	H1-N-H2;H2-N-H3;H3-N-H11
T-v1	−0.3869	−4489.82	3.12	1.01; 1.01; 1.01	106.05; 106.06; 106.01
T-v2	−0.3857	−4475.89	3.02	1.01; 1.01; 1.01	106.94; 106.66; 105.89
B-v1	−0.3869	−4489.82	3.12	1.01; 1.01; 1.01	106.05; 106.05; 106.01
B-v2	−0.3955	−4589.61	3.02	1.01; 1.01; 1.01	106.21; 106.18; 106.09
C-v1	−0.3868	−4488.65	3.10	1.01; 1.01; 1.01	106.07; 106.03; 106.05
C-v2	−0.3319	−3851.56	3.40	1.01; 1.01; 1.01	108.85; 106.78; 106.46

**FIGURE 5:**
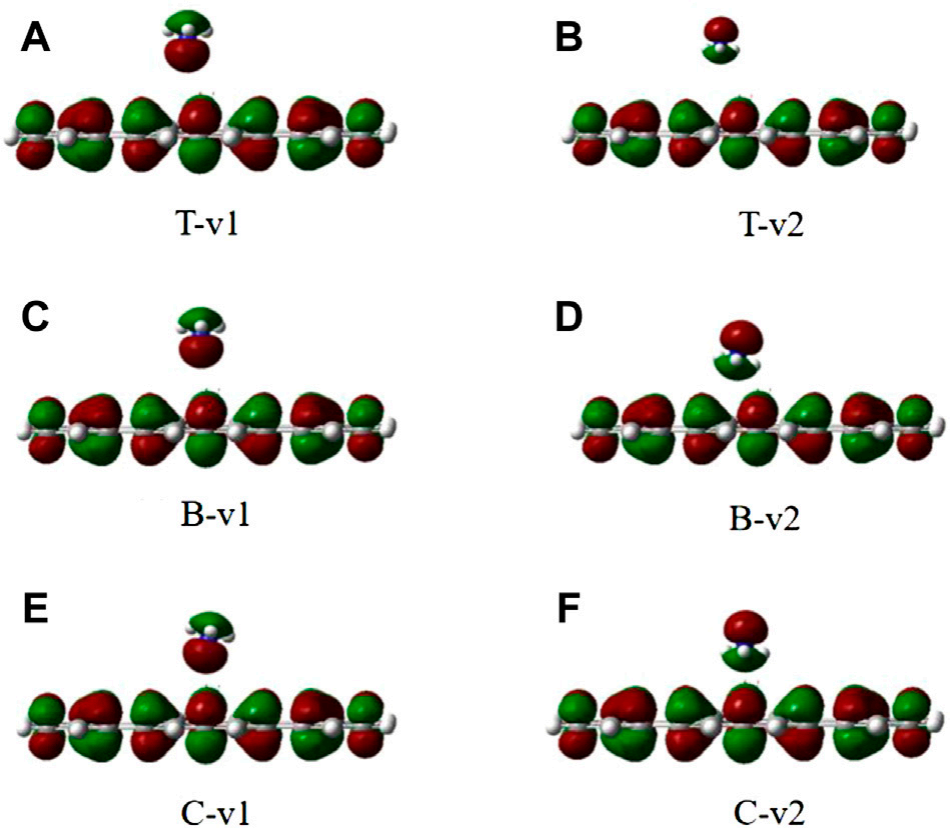
The electron density of six different configurations of NH3 on the graphene-like surface. **(A)**: T-v1, **(B)**: T-v2, **(C)**: B-v1, **(D)**: B-v2, **(E)**: C-v1, **(F)**: and C-v2.

It can be seen from Table 2, when the N atom points to the graphene-like surface, the adsorption energies and the distances to the T, B, and C binding sites are almost the same. When the H atom points to the graphene-like, the order of adsorption energies is B-v2 > T-v2 > C-v2. The main factor affecting the adsorption energy is the adsorption position. Table 2 shows that all the adsorption energies are less than 0.8 eV, and the optimal adsorption configuration is H atoms point to the graphene-like surface. This is because the atomic radius of H atom is smaller than that of other elements, and the repulsive force between H atom and other atoms is smaller. Moreover, the contact area between NH_3_ and the graphene-like surface is the largest in v2 mode, where the hydrogen bond is easily formed between H in NH_3_ and the atom with greater electronegativity (such as C). Therefore the adsorption energy is relatively large. As also shown in Figure 5, it is found that NH_3_ does not overlap with the electron cloud of the graphene-like surface, and the adsorption of NH_3_ is physical adsorption, with no electron transfer.

**FIGURE 6:**
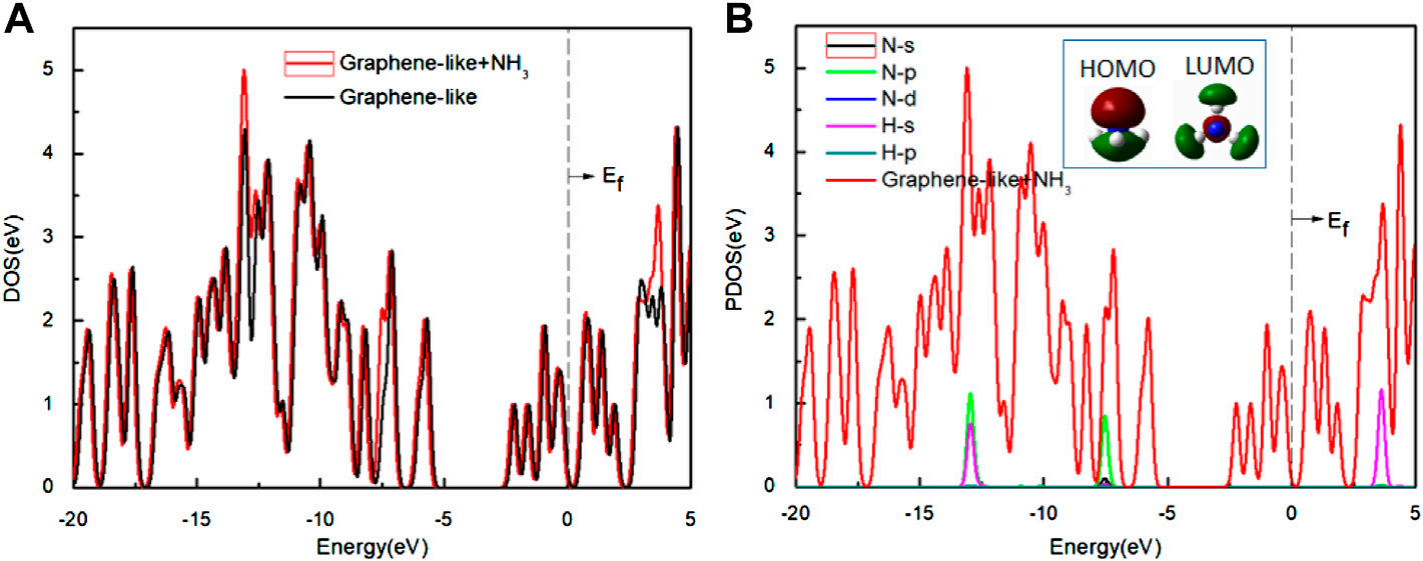
**(A)** The DOS of the graphene-like surface before and after adsorption of NH_3_; **(B)** The PDOS of the NH_3_ on the surface. Inset: HOMO and LUMO of NH_3_ (green and deep red lines indicate different signs of the orbital wavefunctions). Main panel: The red line is the DOS of NH_3_ on the graphene-like surface, and other lines show the PDOS of the H and N atoms, respectively.

We calculated the DOS of the graphene-like surface before and after the adsorption of NH_3_, and show them in Figure 6. In Figure 6A, the TDOS and the pseudogap are almost constant. In Figure 6B, the PDOS of the N and H atoms are almost zero near the Fermi level. Analysis of HOMO and LUMO shows that the electron distribution is concentrated on the C3v symmetry axis of the NH_3_ molecule (concentrated around the N atoms), not on the N-H line. This indicates that the electrons of HOMO are uninvolved in the N-H bonding of the NH_3_ molecule, and that HOMO is occupied by a lone pair of electrons. In LUMO, the electron density is mainly concentrated in the space in between the N and H atoms (near N atom), but not along the N-H bond, indicating that LUMO is in the N-H antibonding position and is unoccupied. Therefore, the electronic properties before and after adsorption are almost unchanged.

### 3.3 CO_2_ on the graphene-like surface

According to the structural characteristics of CO_2_ molecules and the selected binding sites, there are 6 different adsorption configurations of CO_2_ molecules on the graphene-like surface. The calculated adsorption energies of CO_2_ on the graphene-like surface are listed in Table 3.

**TABLE 3:** CO_2_ on the graphene-like surface: *E*_*ads*_, d and the bond length and bond angle of CO_2_.

Configurations	*E*_*ads*_(eV)	*E*_*ads*_(K)	*d*(Å)	Bond length (Å)	Bond angle (°)
				C1-O; C2-O	
T-p	0.3306	3836.48	3.47	1.159; 1.158	179.25
T-v	0.3288	3815.59	3.39	1.159; 1.159	179.33
B-p	0.3288	3815.59	3.39	1.159; 1.159	179.33
B-v	0.3288	3815.59	3.39	1.159; 1.159	179.33
C-p	0.3713	4308.78	3.50	1.160; 1.158	179.99
C-v	0.3288	3815.59	3.39	1.159; 1.159	179.33

**FIGURE 7:**
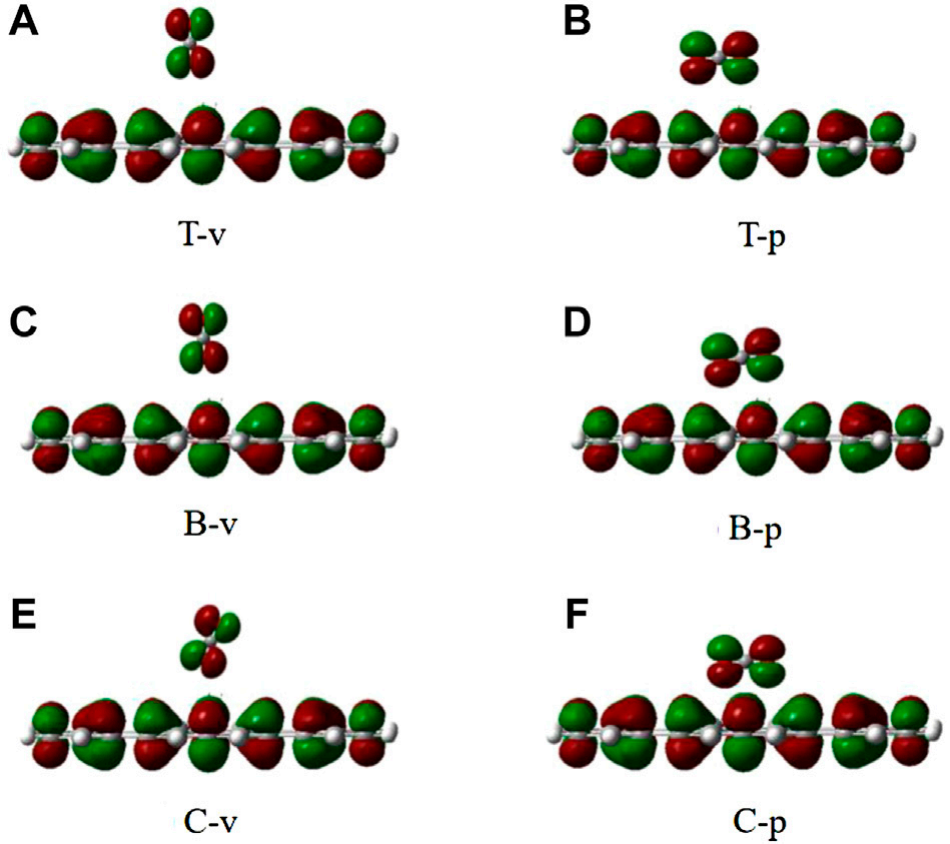
The electron density of six different configurations of CO_2_ on the graphene-like surface. **(A)**: T-v, **(B)**: T-p, **(C)**: B-v, **(D)**: B-p, **(E)**: C-v, **(F)**: and C-p.

As it can be seen from Table 3, all adsorption energies are positive values, indicating that the adsorption of CO_2_ on the graphene-like surface is an endothermic process. The magnitude of adsorption energy mainly depends on the distance. When the distance is the same, the adsorption energy is also the same. The larger the distance, the greater the adsorption energy. Also, the adsorption distances of all modes are greater than 0.34 Å, which is greater than the sum of the covalent bond radii of the corresponding atoms. In combination with the charge density of CO_2_ adsorbed on the graphene-like surface (Figure 7), it can be concluded that CO_2_ molecules do not overlap with the electron cloud of the surface. All adsorption energies are less than 0.8 eV, and the bond length and bond angle are both almost constant, indicating that the adsorption of CO_2_ on the graphene-like surface is weak physical adsorption. The molecular structure has not changed, and there is no electron transfer.

**FIGURE 8:**
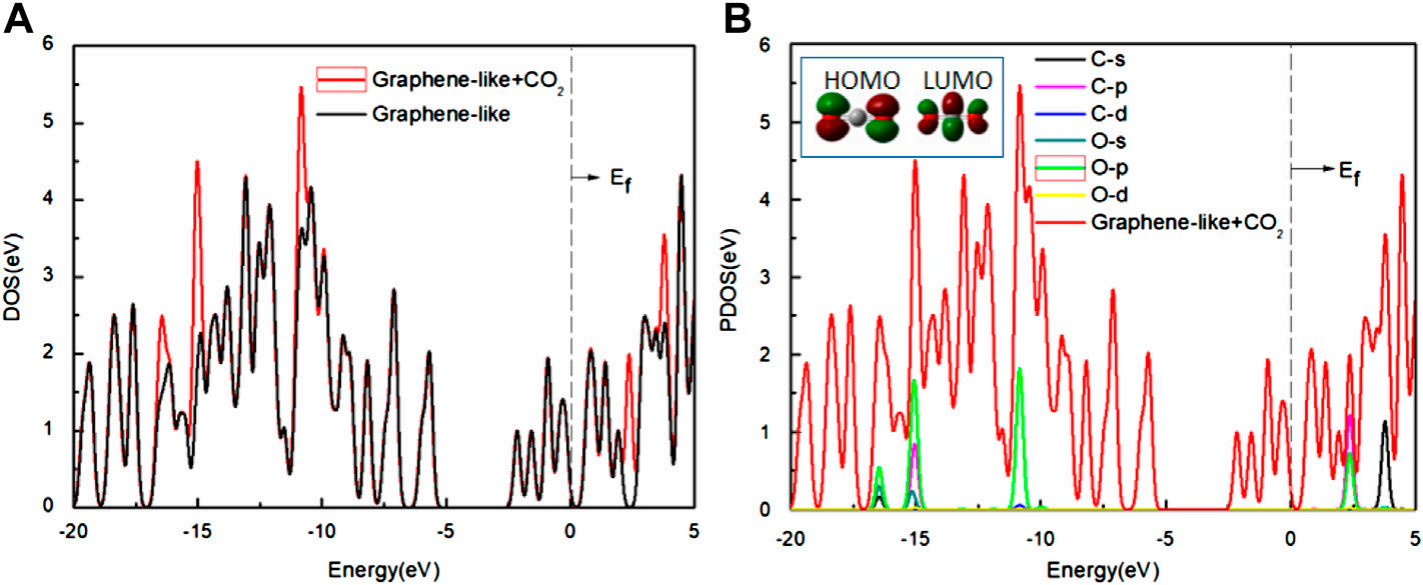
**(A)** The DOS of the graphene-like surface before and after the adsorption of CO_2_; **(B)** The PDOS of the CO_2_ on the surface. Inset: HOMO and LUMO of CO_2_ (green and deep red lines indicate different signs of the orbital wavefunctions). Main panel: The red line is the DOS of CO_2_ on the graphene-like surface, other lines show the PDOS of the various orbits of the C and O atoms, respectively.

We also analyzed the DOS of the graphene-like surface before and after CO_2_ adsorption and show them in Figure 8. From Figure 8A, the TDOS of the graphene-like surface and the pseudogap are unchanged before and after CO_2_ adsorption. No new states appear near the Fermi level, indicating that the adsorption of CO_2_ has no significant effect on the graphene-like surface. Moreover, the near-constant pseudogap in the post-adsorption system shows that CO_2_ has almost no effect on the conductive properties of the system. From the PDOS of Figure 8B, after the CO_2_ adsorption, three independent peaks at the low energy level become larger, and two independent peaks at the high energy level become larger. The positions of these peaks are similar to the PDOS of CO_2_ molecules in Figure 8B. The contribution of electronic levels of CO_2_ after interaction with the graphene-like surface is limited to −16, −15, and −10 eV in the valence band, as well as to 2.5 and 4 eV in the conduction band, which are distant from the Fermi level and cannot change the electronic properties of the graphene-like surface near the Fermi energy (Zahedi and Seif, 2011). It can be concluded that the changes of these peaks correspond to the s and p orbitals of C and O atoms in CO_2_ molecules, respectively.

### 3.4 Discussion

In conclusion, the adsorption energies of H_2_O, NH_3_ and CO_2_ on graphene-like surfaces are all less than 0.8 eV. All these adsorption processes belong to physical adsorption. Table 4 lists some theoretical results from literature. Some of our results differ from the adsorption energies in Table 4, because of the choice of basis set, different substrates and different substrate sizes. (Leenaerts et al., 2008; Zahedi and Seif, 2011; Lalitha et al., 2017; Safari et al., 2019; Li et al., 2022).

**TABLE 4:** Based on the DFT theory, theoretical values for the *E*_*ads*_ of interstellar molecules on different substrates.

Molecules	The *E*_*ads*_ of interstellar molecules on different substrates (eV)			
	Graphene	Blue phosphorene	GaN	C_48_B_6_N_6_
H_2_O	−0.112 ∼−0.160^a^			
NH_3_		−0.132^c^		−0.89^e^
NO		−0.216 ∼−0.223^c^	−0.27^d^	
H	0.22^b^			

^a^ Lalitha et al. (2017).

^b^ Leenaerts et al. (2008).

^c^ Safari et al. (2019).

^d^ Li et al. (2022).

^e^ Zahedi and Seif(2011).

## 4 Summary

We established the stability models of three interstellar molecules (H_2_O, NH_3_, CO_2_) and the graphene-like surface. Based on the DFT, the adsorption energy, adsorption distance, charge density, DOS and other properties of interstellar molecules H_2_O, NH_3,_ and CO_2_ on the graphene-like surface were calculated, and their adsorption properties were studied.

The adsorption of H_2_O, NH_3,_ and CO_2_ on the graphene-like surface belongs to physical adsorption, which is reflected in the following aspects: the absolute value of adsorption energy is less than 0.8 eV, and the bond length and bond angle of H_2_O, NH_3,_ and CO_2_ are almost unchanged.

Moreover, the charge density shows that the gas molecules do not overlap with the base electron cloud. Adsorption of H_2_O, NH_3_, and CO_2_ had little effect on the density of states of the graphene-like surface. The process has no effect on the pseudogap of the surface either. These results show that the electronic properties of the graphene-like surface are unaffected after the adsorption of the studied molecules. They also further verified that the adsorption of the three molecules on the graphene-like surfaces is physical adsorption.

Last but not least, the results can provide more accurate parameters (e.g., binding energies) for calculating the chemical reaction rates on dust surfaces, which in return can provide a more accurate theoretical reference for the models and observations of interstellar molecules, thus contributing to the understanding of dust-surface reactions in ISM.

On the basis of this work, we will further take carbonaceous materials as the core, and use amorphous phase H_2_O and other molecules (such as CO, CO_2_, NH_3_, CH_4_, H_2_CO, and CH_3_OH) as ice mantles to establish a dust particle model, simulate the adsorption of interstellar molecules on the ice surface, and calculate adsorption energies.

## Data Availability

The original contributions presented in the study are included in the article/Supplementary Material, further inquiries can be directed to the corresponding author.

## Footnotes

1
https://cdms.astro.uni-koeln.de/classic/molecules
