# Supplementary material for: Quantum mechanical modeling of interstellar molecules on cosmic dusts: H2O, NH3, and CO2
Source: Front Chem. 2022 Nov 10;10:1040703. doi: 10.3389/fchem.2022.1040703 (PMC9684330; doi:10.3389/fchem.2022.1040703)
Supplement: Supplementary file 1 [file DataSheet1.doc]

**Supplementary Information for**

**Quantum Mechanical Modeling of Interstellar Molecules on Cosmic Dusts: H_2_O, NH_3_, and CO_2_.**

Fangfang Li^1,2^, Donghui Quan^3,4*^, Xia Zhang^1,5^, Xiaohu Li^1,6^, Jarken Esimbek^1,6^

^1^Xinjiang Astronomical Observatory, Chinese Academy of Sciences, Urumqi 830011, China

^2^School of Astronomy and Space Science, University of the Chinese Academy of Sciences, Beijing 100049, China

^3^Research Center for Intelligent Computing Platforms, Zhejiang Laboratory, Hangzhou 311100, China

^4^Department of Chemistry, Eastern Kentucky University, Richmond, KY 40475, USA

^5^Xinjiang Key Laboratory of Radio Astronomy, Urumqi 830011, China

^6^Key Laboratory of Radio Astronomy, Chinese Academy of Sciences, Urumqi 830011, China

*** Correspondence: Donghui Quan**

Corresponding Author

quandh@xao.ac.cn

**Contents**

Table S1 The xyz coordinates of the clean surface without adsorption

Table S2 The xyz coordinates of surface with H_2_O adsorption

Table S3 The xyz coordinates of surface with NH_3_ adsorption

Table S4 The xyz coordinates of surface with CO_2_ adsorption

Table S1 The xyz coordinates of the clean surface without adsorption

| atom | x | y | z |
| --- | --- | --- | --- |
| C | -3.867038 | 1.485191 | -0.000553 |
| C | -5.023545 | 0.736483 | 0.000748 |
| C | -3.861153 | 2.894736 | -0.000403 |
| C | -5.009033 | 3.639663 | 0.001049 |
| C | -6.206314 | 1.511958 | 0.002403 |
| C | -6.201033 | 2.895308 | 0.002514 |
| C | 2.370823 | 1.477378 | -0.001682 |
| C | 1.231958 | 0.730651 | -0.001238 |
| C | 2.368827 | 2.897282 | -0.001672 |
| C | 1.228133 | 3.624014 | -0.000997 |
| C | -0.000004 | 1.467229 | -0.001207 |
| C | -1.231957 | 0.730649 | -0.001271 |
| C | -0.000005 | 2.896471 | -0.001048 |
| C | -1.228133 | 3.624011 | -0.001015 |
| C | -2.370826 | 1.477377 | -0.001832 |
| C | -2.368827 | 2.897278 | -0.001736 |
| C | 6.206311 | 1.51196 | 0.002185 |
| C | 5.023543 | 0.736485 | 0.000724 |
| C | 6.201029 | 2.895311 | 0.002206 |
| C | 5.00903 | 3.639666 | 0.000841 |
| C | 3.867034 | 1.485193 | -0.000507 |
| C | 3.861151 | 2.894737 | -0.000426 |
| C | -3.861148 | -2.894721 | 0.000125 |
| C | -3.867026 | -1.485161 | 0.000078 |
| C | -5.02353 | -0.736489 | 0.000371 |
| C | -5.008996 | -3.639679 | 0.000275 |
| C | -6.201021 | -2.895324 | 0.000268 |
| C | -6.206313 | -1.511993 | 0.000301 |
| C | 2.368835 | -2.897235 | -0.000103 |
| C | 2.370811 | -1.477378 | -0.000147 |
| C | 1.231914 | -0.730615 | -0.000634 |
| C | 1.228091 | -3.624057 | -0.000323 |
| C | 0.000001 | -2.89655 | -0.000453 |
| C | 0.000001 | -1.467136 | -0.000561 |
| C | -1.231911 | -0.730616 | -0.000644 |
| C | -1.228088 | -3.624058 | -0.00035 |
| C | -2.368833 | -2.897236 | -0.000121 |
| C | -2.370807 | -1.47738 | -0.000138 |
| C | 6.201024 | -2.895322 | 0.00048 |
| C | 6.206315 | -1.51199 | 0.000466 |
| C | 5.023531 | -0.736488 | 0.000434 |
| C | 5.009 | -3.639678 | 0.000442 |
| C | 3.86115 | -2.894721 | 0.000224 |
| C | 3.867029 | -1.485162 | 0.000136 |
| H | 7.166402 | 1.014557 | 0.003338 |
| H | 7.149814 | 3.416496 | 0.00333 |
| H | -5.020852 | -4.721046 | 0.000238 |
| H | 1.205997 | -4.706197 | -0.000259 |
| H | -1.205995 | -4.706198 | -0.000314 |
| H | 7.14979 | -3.416543 | 0.000471 |
| H | 7.166397 | -1.014572 | 0.000419 |
| H | 5.020857 | -4.721045 | 0.000432 |
| H | 5.020879 | 4.721033 | 0.000938 |
| H | 1.205952 | 4.706163 | -0.000456 |
| H | -1.205956 | 4.706158 | -0.000461 |
| H | -5.020884 | 4.72103 | 0.001235 |
| H | -7.149818 | 3.416492 | 0.00379 |
| H | -7.166405 | 1.014555 | 0.00367 |
| H | -7.166394 | -1.014574 | 0.00019 |
| H | -7.149787 | -3.416547 | 0.000184 |

Table S2 The xyz coordinates of surface with H_2_O adsorption

| atom | x | y | z |
| --- | --- | --- | --- |
| C | 3.867135 | 1.294224 | -0.159366 |
| C | 5.02366 | 0.546865 | -0.113972 |
| C | 3.861111 | 2.701025 | -0.246215 |
| C | 5.008707 | 3.445112 | -0.292316 |
| C | 6.20607 | 1.321267 | -0.161465 |
| C | 6.2006 | 2.702156 | -0.246465 |
| C | -2.370961 | 1.28579 | -0.160266 |
| C | -1.232207 | 0.540063 | -0.117293 |
| C | -2.368641 | 2.703401 | -0.246805 |
| C | -1.228312 | 3.430618 | -0.294818 |
| C | -0.000023 | 1.275423 | -0.163714 |
| C | 1.232224 | 0.54019 | -0.117196 |
| C | -0.000116 | 2.701461 | -0.252586 |
| C | 1.228129 | 3.430618 | -0.295339 |
| C | 2.37098 | 1.285974 | -0.159898 |
| C | 2.368585 | 2.703519 | -0.246863 |
| C | -6.206065 | 1.320784 | -0.161166 |
| C | -5.023575 | 0.546485 | -0.114037 |
| C | -6.200723 | 2.701687 | -0.245917 |
| C | -5.008889 | 3.444737 | -0.291805 |
| C | -3.867104 | 1.293939 | -0.159529 |
| C | -3.86119 | 2.700765 | -0.246044 |
| C | 3.861549 | -3.077796 | 0.105848 |
| C | 3.867355 | -1.670888 | 0.020033 |
| C | 5.023804 | -0.923408 | -0.024345 |
| C | 5.009469 | -3.821222 | 0.152295 |
| C | 6.201366 | -3.078035 | 0.108224 |
| C | 6.206622 | -1.697223 | 0.024092 |
| C | -2.368843 | -3.080793 | 0.104593 |
| C | -2.370891 | -1.663575 | 0.018089 |
| C | -1.231912 | -0.918427 | -0.028146 |
| C | -1.227981 | -3.80616 | 0.14841 |
| C | 0.000164 | -3.080025 | 0.103789 |
| C | 0.000137 | -1.653368 | 0.01643 |
| C | 1.232107 | -0.918308 | -0.028084 |
| C | 1.228373 | -3.806039 | 0.148553 |
| C | 2.369196 | -3.08059 | 0.104789 |
| C | 2.371139 | -1.66338 | 0.018216 |
| C | -6.201017 | -3.078539 | 0.107541 |
| C | -6.206369 | -1.697717 | 0.023542 |
| C | -5.023603 | -0.923809 | -0.024664 |
| C | -5.009074 | -3.821637 | 0.151706 |
| C | -3.861192 | -3.078134 | 0.105424 |
| C | -3.867107 | -1.671223 | 0.019715 |
| H | -7.166249 | 0.824514 | -0.130896 |
| H | -7.149468 | 3.221882 | -0.278026 |
| H | 5.021465 | -4.900568 | 0.218044 |
| H | -1.205874 | -4.886274 | 0.214344 |
| H | 1.206365 | -4.886155 | 0.21453 |
| H | -7.149776 | -3.598717 | 0.139988 |
| H | -7.166479 | -1.201245 | -0.005789 |
| H | -5.021007 | -4.900991 | 0.217369 |
| H | -5.020459 | 4.523991 | -0.359339 |
| H | -1.199295 | 4.51059 | -0.354425 |
| H | 1.199394 | 4.510539 | -0.357024 |
| H | 5.02014 | 4.524355 | -0.359934 |
| H | 7.149291 | 3.222435 | -0.278766 |
| H | 7.166299 | 0.825078 | -0.131257 |
| H | 7.166693 | -1.200669 | -0.005157 |
| H | 7.15016 | -3.598135 | 0.140841 |
| O | 0.001059 | 5.394329 | 1.963427 |
| H | -0.764512 | 4.830027 | 2.068158 |
| H | 0.738711 | 4.789852 | 2.042072 |

Table S3 The xyz coordinates of surface with NH3 adsorption

| atom | x | y | z |
| --- | --- | --- | --- |
| C | 3.866948 | 1.488621 | -0.114214 |
| C | 5.023356 | 0.73972 | -0.11458 |
| C | 3.860963 | 2.898022 | -0.110967 |
| C | 5.008759 | 3.643123 | -0.108279 |
| C | 6.205923 | 1.515204 | -0.111888 |
| C | 6.200527 | 2.898663 | -0.108931 |
| C | -2.371842 | 1.480719 | -0.114218 |
| C | -1.232866 | 0.73414 | -0.115961 |
| C | -2.369719 | 2.9008 | -0.110675 |
| C | -1.228999 | 3.62759 | -0.10862 |
| C | -0.000546 | 1.470796 | -0.11458 |
| C | 1.23182 | 0.734137 | -0.116335 |
| C | -0.000545 | 2.90016 | -0.110973 |
| C | 1.227919 | 3.627587 | -0.109078 |
| C | 2.370818 | 1.480725 | -0.114958 |
| C | 2.368666 | 2.90081 | -0.111541 |
| C | -6.206973 | 1.515162 | -0.110578 |
| C | -5.024397 | 0.739687 | -0.113463 |
| C | -6.201583 | 2.89862 | -0.107556 |
| C | -5.009815 | 3.643083 | -0.107022 |
| C | -3.867984 | 1.488589 | -0.113163 |
| C | -3.86201 | 2.897995 | -0.109855 |
| C | 3.861023 | -2.891638 | -0.119308 |
| C | 3.867054 | -1.482215 | -0.117449 |
| C | 5.023404 | -0.73323 | -0.116928 |
| C | 5.008821 | -3.636744 | -0.12084 |
| C | 6.200561 | -2.892231 | -0.12027 |
| C | 6.205949 | -1.508761 | -0.118447 |
| C | -2.369722 | -2.894463 | -0.118685 |
| C | -2.371939 | -1.474265 | -0.116415 |
| C | -1.232792 | -0.727484 | -0.117531 |
| C | -1.228892 | -3.621044 | -0.12153 |
| C | -0.000532 | -2.893438 | -0.121702 |
| C | -0.000548 | -1.464338 | -0.120018 |
| C | 1.231738 | -0.727475 | -0.117878 |
| C | 1.227834 | -3.621036 | -0.121795 |
| C | 2.368701 | -2.894464 | -0.119318 |
| C | 2.370903 | -1.474259 | -0.117122 |
| C | -6.201591 | -2.892276 | -0.119366 |
| C | -6.206997 | -1.508803 | -0.117451 |
| C | -5.024454 | -0.73327 | -0.115943 |
| C | -5.009849 | -3.63678 | -0.120048 |
| C | -3.862042 | -2.891678 | -0.118557 |
| C | -3.868099 | -1.482259 | -0.116633 |
| H | -7.167109 | 1.017922 | -0.110184 |
| H | -7.150344 | 3.419753 | -0.105086 |
| H | 5.020748 | -4.718063 | -0.122109 |
| H | -1.206762 | -4.703127 | -0.123146 |
| H | 1.20569 | -4.70312 | -0.123299 |
| H | -7.150359 | -3.413402 | -0.120113 |
| H | -7.16714 | -1.011582 | -0.116917 |
| H | -5.021775 | -4.718099 | -0.121374 |
| H | -5.021727 | 4.724401 | -0.104069 |
| H | -1.206994 | 4.709674 | -0.105247 |
| H | 1.205916 | 4.709672 | -0.105689 |
| H | 5.020666 | 4.724441 | -0.105373 |
| H | 7.149286 | 3.419799 | -0.106597 |
| H | 7.166064 | 1.017974 | -0.111561 |
| H | 7.166083 | -1.011525 | -0.117967 |
| H | 7.149332 | -3.413349 | -0.121028 |
| N | 0.015807 | -0.089265 | 3.341472 |
| H | 0.082881 | 0.837287 | 2.943364 |
| H | -0.828262 | -0.495969 | 2.962466 |
| H | 0.781437 | -0.618633 | 2.947651 |

Table S4 The xyz coordinates of surface with CO_2_ adsorption

| atom | x | y | z |
| --- | --- | --- | --- |
| C | -3.901889 | -1.407313 | -0.289243 |
| C | -5.064915 | -0.670826 | -0.233729 |
| C | -3.883253 | -2.814276 | -0.371477 |
| C | -5.024198 | -3.569185 | -0.402926 |
| C | -6.240382 | -1.45653 | -0.266637 |
| C | -6.222661 | -2.837521 | -0.347198 |
| C | 2.335822 | -1.340078 | -0.34592 |
| C | 1.190277 | -0.604642 | -0.293551 |
| C | 2.346315 | -2.757704 | -0.430293 |
| C | 1.212449 | -3.49392 | -0.464475 |
| C | -0.035728 | -1.352308 | -0.326961 |
| C | -1.27422 | -0.628212 | -0.270642 |
| C | -0.022269 | -2.779461 | -0.410318 |
| C | -1.244008 | -3.517467 | -0.44043 |
| C | -2.40601 | -1.384894 | -0.304055 |
| C | -2.391001 | -2.802501 | -0.387059 |
| C | 6.170854 | -1.337887 | -0.385062 |
| C | 4.981444 | -0.574921 | -0.32807 |
| C | 6.17776 | -2.718985 | -0.465878 |
| C | 4.992755 | -3.473529 | -0.497992 |
| C | 3.83194 | -1.333756 | -0.360479 |
| C | 3.838367 | -2.740764 | -0.443122 |
| C | -3.935211 | 2.965151 | -0.034367 |
| C | -3.928409 | 1.557963 | -0.11611 |
| C | -5.078132 | 0.79962 | -0.148298 |
| C | -5.089711 | 3.697736 | 0.020001 |
| C | -6.274962 | 2.943295 | -0.011736 |
| C | -6.267863 | 1.562324 | -0.091981 |
| C | 2.294197 | 3.027378 | -0.093508 |
| C | 2.308929 | 1.610064 | -0.176118 |
| C | 1.176672 | 0.854197 | -0.208425 |
| C | 1.146902 | 3.741803 | -0.040463 |
| C | -0.074541 | 3.003855 | -0.070932 |
| C | -0.061777 | 1.57708 | -0.153572 |
| C | -1.286776 | 0.830333 | -0.184594 |
| C | -1.308991 | 3.718387 | -0.017177 |
| C | -2.443096 | 2.981961 | -0.048424 |
| C | -2.432278 | 1.564598 | -0.13067 |
| C | 6.126211 | 3.061614 | -0.126841 |
| C | 6.143997 | 1.680791 | -0.207278 |
| C | 4.968127 | 0.895529 | -0.242399 |
| C | 4.927554 | 3.793428 | -0.073469 |
| C | 3.786405 | 3.039058 | -0.107047 |
| C | 3.804979 | 1.63192 | -0.189162 |
| H | 7.126459 | -0.832316 | -0.365647 |
| H | 7.131122 | -3.230206 | -0.505452 |
| H | -5.111309 | 4.777104 | 0.083094 |
| H | 1.115015 | 4.821828 | 0.022946 |
| H | -1.296627 | 4.798845 | 0.045584 |
| H | 7.070193 | 3.590971 | -0.104507 |
| H | 7.108446 | 1.193297 | -0.244145 |
| H | 4.929787 | 4.873018 | -0.010524 |
| H | 5.014214 | -4.552854 | -0.561078 |
| H | 1.200113 | -4.574299 | -0.525862 |
| H | -1.212059 | -4.597491 | -0.502599 |
| H | -5.02628 | -4.648776 | -0.465806 |
| H | -7.166668 | -3.366897 | -0.367692 |
| H | -7.204878 | -0.969296 | -0.227682 |
| H | -7.223401 | 1.056604 | -0.111644 |
| H | -7.228302 | 3.454577 | 0.027695 |
| C | 0.616318 | -1.429015 | 3.036421 |
| O | 0.189435 | -0.351776 | 3.0507 |
| O | 1.042842 | -2.506778 | 3.037215 |
